# Neutrophil TLR4 and PKR are targets of breast cancer cell glycosaminoglycans and effectors of glycosaminoglycan-induced APRIL secretion

**DOI:** 10.1038/s41389-018-0058-2

**Published:** 2018-06-15

**Authors:** Uilst Bat-Erdene, Eric Quan, Kelvin Chan, Brianna-Marie Lee, Wejdan Matook, Ki-Young Lee, Jesusa L. Rosales

**Affiliations:** 1 Snyder Institute for Chronic Diseases, Department of Biochemistry and Molecular Biology, University of Calgary, Calgary, AB T2N 4N1 Canada; 2 Arnie Charbonneau Cancer Institute, Department of Cell Biology and Anatomy, University of Calgary, Calgary, AB T2N 4N1 Canada

## Abstract

A proliferation-inducing ligand (APRIL), which induces survival and migration signals and tumor growth, is commonly observed in breast cancer tissues but is not often expressed in breast cancer cells themselves. Here, we examined whether breast cancer cells induce APRIL secretion from neutrophils, which are frequently recruited into the breast tumor microenvironment. We found that breast cancer cells do stimulate neutrophils to secrete APRIL through their glycosaminoglycans. Breast cancer cells depleted of heparan sulfate or chondroitin sulfate glycosaminoglycans lose their ability to induce APRIL secretion from neutrophils, and heparan sulfate and chondroitin sulfate can induce secretion that is comparable to that of breast cancer cell-induced secretion. While stimulation of the RNA-activated protein kinase (PKR) is sufficient to induce neutrophil APRIL secretion, both PKR and the toll-like receptor 4 (TLR4) are required for breast cancer cell glycosaminoglycan-induced secretion as separate and specific inhibition of TLR4 or PKR completely prevents the process, suggesting that breast cancer cell glycosaminoglycans target neutrophil TLR4 and PKR to trigger APRIL secretion. Thus, apart from the putative role of cell surface heparan sulfate in binding APRIL that leads to cell growth, we demonstrate that heparan sulfate, as well as chondroitin sulfate plays a novel role in promoting neutrophil secretion of APRIL that could lead to further cell growth. We propose that breast cancer cells take advantage of the neutrophil recruitment to the tumor microenvironment through the dual role of heparan sulfate as cell surface receptor or docking molecule for APRIL and as a ligand that induces neutrophil APRIL secretion to promote their own growth.

## Introduction

Cytokines have been implicated in cancer initiation and progression^1,2^. For example, the cytokine ‘a proliferation-inducing ligand’ (APRIL), stimulates survival and migration signals^3^ and tumor growth^4^, although, it was also determined to modulate death ligand-induced apoptosis^5^. The encoded 250 amino acid APRIL protein^4^ is cleaved in the Golgi at an arginine-rich motif by furin convertase to produce an 11 kDa stalk that remains in the cell and a 16 kDa soluble active form that is secreted extracellularly^6^. Secreted APRIL serves as a ligand for the tumor necrosis factor (TNF) receptors, transmembrane activator and calcium-modulator and cyclophilin ligand (CAML)-interactor (TACI) and B-cell maturation antigen (BCMA)^7^. However, APRIL also binds specifically to the heparan sulfate (HS) side chains of heparan sulfate proteoglycans (HSPGs)^8,9^, facilitating APRIL-induced functions^3,8^. This led to the suggestion that HSPG may act as an APRIL receptor or docking site that permits APRIL interaction with a receptor^8^.

Analysis of publicly available Oncomine Cancer Microarray and Amazonia gene expression databases revealed APRIL overexpression in 1 out of 4 different types of hematological malignancies and in 6 out of 36 different types of solid tumors^10^. Multi-tumor tissue microarrays also revealed increased APRIL levels in some solid tumors but stromal neutrophils were found to be the main source of APRIL in these tumors^11^. In fact, out of 2159 tumors examined, only 20 (0.9%) showed tumor cells as the sole source of APRIL^11^ and thus APRIL overexpression in tumors was suggested to result from neutrophil infiltration^10^. This is consistent with a role for neutrophils in tumor progression^12^.

Neutrophils have long been established as major players and the first line of defense in the innate immune system. Their role in carcinogenesis, however, is still under deliberation^13–15^. Traditionally, neutrophils were thought to inhibit tumor growth and promote tumor regression through recruitment of tumor-specific cytotoxic T cells^16,17^. However, it is now clear that neutrophils also serve to promote cancer progression^18^ and new insights on their direct and indirect effects on cancer cells and the tumor microenvironment continue to emerge. For instance, neutrophils cooperate with IL-17-producing γδ T cells to promote breast cancer metastasis^19^. Certainly, even with a normally functioning immune system, tumors grow and develop mechanisms to amplify their growth and metastasize. Breast tumor cells, for example, produce neutrophil mobilizing factors, such as granulocyte colony-stimulating factor that allow tumor stroma infiltration by neutrophils^20,21^, which can stimulate breast tumor progression^13,22^. We (unpublished observation) and others^23^ also found that APRIL, which exists in neutrophils in the breast tumor stroma^11^, promotes growth of breast tumor cells. While APRIL is also recognized to be expressed in other immune cells, including dendritic cells and some lymphocytes, APRIL detection in breast cancer cells has been inconsistent. For example, while Mhawech-Fauceglia et al.^11^ found that only 0.7% of breast tumor samples examined was positive for APRIL in tumor cells themselves, García-Castro et al.^23^ demonstrated the presence of APRIL in breast cancer cell lines. In cases where APRIL was found in breast tumor cells themselves^11,24^, expression was inversely related to tumor grade, and immunoreactivity was lower in malignant compared to non-malignant cells^24^. Why malignant transformation correlates with reduced APRIL expression is unknown and warrants investigation, particularly because APRIL is associated with breast cancer cell (BCC) growth. We then wondered whether BCCs have a mechanism to exploit APRIL from neutrophils in the tumor stroma.

In this study, we show that BCCs induce APRIL secretion from neutrophils, and this toll-like receptor 4 (TLR4)- and RNA-activated protein kinase (PKR)-mediated process is activated by HS and chondroitin sulfate (CS) glycosaminoglycans (GAGs) in BCCs. Thus, BCCs may utilize their HS not only as a cell surface receptor or docking molecule for APRIL, which can promote their growth^23^, but also as a ligand that can induce secretion of neutrophil APRIL, which can then further enhance their growth. Our findings indicate a novel mechanism whereby BCCs can take advantage of the neutrophil immune response and recruitment to tumor tissues for their own growth.

## Results and discussion

### Breast cancer cells induce APRIL secretion from neutrophils

**Fig. 1:**
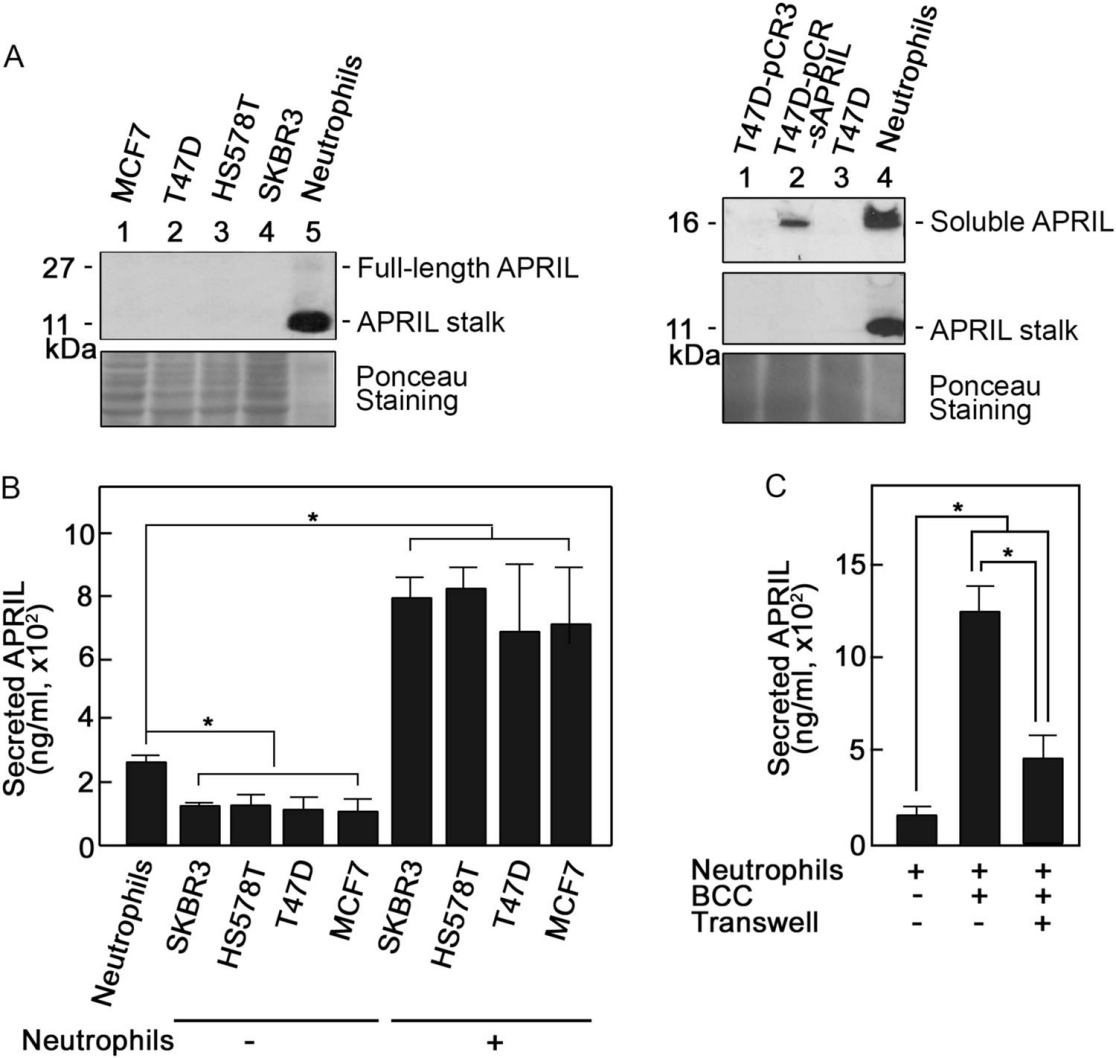
Breast cancer cells induce secretion of APRIL from neutrophils. Following approval by the University of Calgary’s Conjoint Health Research Ethics Board, blood was obtained from healthy volunteers that gave informed consent. Neutrophils were isolated as described previously^48,49^ and suspended in Hank’s Balanced Salt Solution with Ca^2+^/Mg^2+^ (Gibco). BCCs (Hs578T, SKBR3, T47D, and MCF7) obtained from the American Type Culture Collection (not authenticated but tested for mycoplasma in the current year) were cultured in Dulbecco’s Modified Eagle Medium (Gibco) with 10% fetal bovine serum (Gibco) and 100 U/ml each of penicillin and streptomycin (Gibco) in 5% CO_2_ at 37 °C. **a** APRIL stalk is clearly detectable in neutrophils but not in breast cancer cells. On the left panel, lysates of MCF7, T47D, Hs578T, and SKBR3 cells (60 μg each), as well as neutrophils (5 μg) were subjected to SDS-PAGE and western blotting using APRIL stalk antibody (ED, MyBioSource: MBS194153). On the right panel, neutrophils and T47D cells [non-transfected and transfected with pCR3 (T47D-pCR3) or pCR3-soluble APRIL (T47D-pCR3-sAPRIL)] were immunoblotted using Aprily-5 (Novus Biologicals: NBP1-97599; top panel) and reblotted using April ED (middle panel) antibodies. Aprily-5 detects soluble APRIL. The bottom panel shows Ponceau S staining of the membranes. **b** Breast cancer cells enhance APRIL secretion from neutrophils. Neutrophils (2 × 10^6^) and breast cancer cells (5 × 10^5^) were incubated alone or together. Cells were then pelleted and supernatants were analyzed for secreted neutrophil APRIL by direct ELISA. Recombinant APRIL (Alexis Corp.) was used as standard. Anti-APRIL (H-60, Santa Cruz Biotech.:sc-28919) used as detection antibody was complexed with HRP-linked anti-rabbit IgG (Cell Signaling). Immunoreactivity was detected using 3,3′,5,5′-tetramethylbenzidine liquid substrate (Sigma). The colorimetric reaction was stopped using 2 M HCl and measured at 490 nm using a SpectraMax M2/M2e multi-mode microplate reader. Measured APRIL in 1 ml corresponds to secretion from 1 × 10^6^ neutrophils. Note that measured levels of APRIL from 5 × 10^5^ and 2 × 10^6^ breast cancer cells were similar and reflect background values. **c** Breast cancer cell-associated and -released factors are involved in stimulating neutrophil APRIL secretion. Neutrophils (2 × 10^6^) and T47D BCCs (5 × 10^5^) were co-incubated in the presence or absence of transwell membranes (3 μm pore size; Corning) in 24-well plates. When transwell membranes were used, BCCs were loaded in the upper chamber and neutrophils in the lower chamber. Following incubation for 5 min at 37 °C, cells were pelleted and supernatants were analyzed for secreted neutrophil APRIL by ELISA as described above. Values are means ± SD of three replicates of a representative experiment (*n* = 3). One-tailed Student’s *t*-test was used to determine statistical significance at *p* < 0.05. Note that neutrophils used in independent experiments were isolated from different individuals and thus the variation in constitutive/base levels of APRIL secretion and the extent of secretion following stimulation. The specificity of measured APRIL secretion was confirmed using the soluble APRIL blocking peptide, BP1^50^ (data not shown)

We began our studies by analyzing APRIL expression in neutrophils and in four different BCC models: the claudin-low (ER^−^, PR^−^, and HER2^−^) Hs578T; the HER2 (ER^−^, PR^−^, and HER2^+^) SKBR3; and the luminal A (ER^+^, PR^+^, and HER2^−^) T47D and MCF7 cells. Figure 1a shows that while APRIL stalk was clearly observed in neutrophils (top left and middle right panels), it was not detected in MCF7, T47D, Hs578T, and SKBR3 BCCs. A faint 27 kDa band corresponding to full-length APRIL was also detected in neutrophils (top left panel). Soluble APRIL was also detected in neutrophils and in T47D cells transfected with soluble APRIL but not in T47D cells that were untransfected or transfected with vector alone (top right panel). APRIL stalk was not detected in soluble APRIL-transfected T47D cells, indicating antibody specificity. Our observations are consistent with a report indicating that APRIL is not often expressed in BCCs themselves^11^. Indeed, of 130 breast tumors examined, only 1 was positive for APRIL in tumor cells themselves, 50 were positive for APRIL in the tumor stroma, none had positive APRIL staining in both tumor cells and stroma, and 79 had no APRIL staining in both tumor cells and stroma^11^. Discrepancy from the finding of García-Castro et al.^23^ may be explained by different experimental systems.

Nonetheless, given that APRIL is often present in breast cancer tissues and neutrophils are the main source of APRIL in these tissues^11^, we examined the possibility that BCCs induce APRIL secretion from neutrophils. We found that neutrophils constitutively secrete a certain level of APRIL (Fig. 1b, first bar) but co-incubation with BCCs caused a significant increase in neutrophil APRIL secretion (Fig. 1b), suggesting that BCCs stimulate neutrophils to secrete APRIL. This finding concurs with the observed APRIL in breast tumor stromas infiltrated with neutrophils^11^. To identify potential BCC factors that cause stimulation of neutrophil APRIL secretion, we co-incubated neutrophils and T47D BCCs in the presence or absence of a transwell membrane, which prevented direct contact between these cells but allowed the exchange of soluble/secreted factors through the transwell pores. As shown in Fig. 1c, co-incubation of neutrophils with BCCs in the presence or absence of a transwell membrane both resulted in a significant increase in neutrophil APRIL secretion but secretion is substantially greater in the absence of a transwell membrane. These findings suggest that both BCC-associated and -released extracellular factors are involved in inducing neutrophil APRIL secretion.

### Breast cancer cell heparan sulfate and chondroitin sulfate glycosaminoglycans induce APRIL secretion from neutrophils

**Fig. 2:**
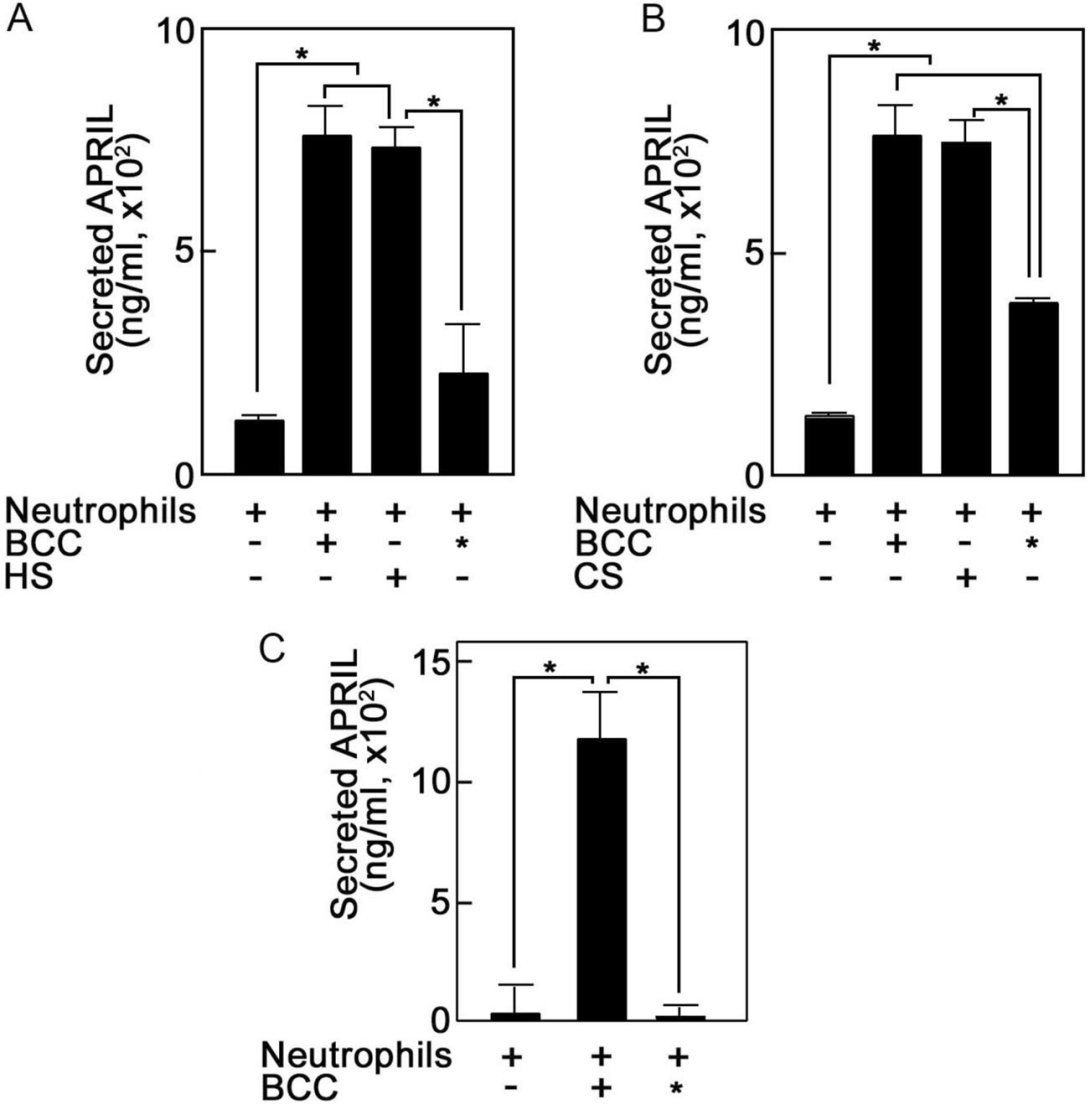
Breast cancer cell heparan sulfate and chondroitin sulfate glycosaminoglycans induce APRIL secretion from neutrophils. **a** Breast cancer cell heparan sulfate induces APRIL secretion from neutrophils. Neutrophils (2 × 10^6^) were incubated alone or with HS (10 μg/mL; Sigma) or T47D BCCs (5 × 10^5^) that were either untreated or treated with heparinase II (0.2 U/mL; Sigma) for 7 days in culture. * denotes BCCs depleted of HS by heparinase II treatment. **b** Breast cancer cell chondroitin sulfate induces APRIL secretion from neutrophils. Neutrophils (2 × 10^6^) were incubated alone or with CS (20 μg/mL; Sigma) or BCCs (5 × 10^5^) that were either untreated or treated with chondroitinase ABC (50 mU/mL; Sigma) for 7 days in culture. * denotes BCCs depleted of CS by chondroitinase treatment. **c** Glycosaminoglycan sulfation is required to induce neutrophil APRIL secretion. Neutrophils (2 × 10^6^) were incubated alone or with BCCs (5 × 10^5^) that were either untreated or treated with sodium chlorate (50 mM; Sigma) for 7 days in culture. * denotes BCCs depleted of GAG sulfation by sodium chlorate treatment. BCCs in culture were given fresh media with or without added treatments every 2 days. Neutrophils were stimulated with BCCs, HS, or CS for 5 min at 37 °C. Following centrifugation, supernatants containing secreted neutrophil APRIL were analyzed by ELISA as described in Fig. legend 1. Values are means ± SD from three replicates of a representative experiment; *n* = 5. Statistical significance was determined using one-tailed Student’s *t*-test (*p* < 0.05)

Since HS GAGs exist as free chains or as a component of proteoglycans in both the cell surface and extracellularly, we examined the possibility that BCC HS induces neutrophil APRIL secretion. Figure 2a shows that BCCs depleted of HS (*) by heparinase treatment have reduced ability to induce neutrophil APRIL secretion, and HS-induced secretion to an extent similar to that induced by BCCs that are not depleted of HS, suggesting that BCC HS stimulates neutrophil APRIL secretion. Since HS depletion did not completely inhibit BCC-induced secretion, we examined the possibility that CS, the other major GAG on the cell surface that is also released extracellularly, contributes to BCC stimulation of neutrophil APRIL secretion. As shown in Fig. 2b, BCCs depleted of CS (*) by chondroitinase treatment reduced the ability of BCCs to induce secretion, and as with HS, CS induced neutrophil APRIL secretion to an extent similar to that induced by BCCs that are not depleted of CS or HS, suggesting that CS together with HS contributes to BCC stimulation of neutrophil APRIL secretion. The similar extents of induced APRIL secretion by BCC, HS, and CS may indicate that maximal APRIL secretion has been reached with the amounts of neutrophils, BCCs and GAGs used in the experiments. Together, the above findings indicate a new functional role for GAGs as inducers of neutrophil APRIL secretion.

Since sulfation is a major biosynthetic GAG modification, we next examined its importance in GAG-induced neutrophil APRIL secretion. Figure 2c shows that inhibition of GAG sulfation by sodium chlorate treatment^25^ (*) completely inhibited the ability of BCCs to induce neutrophil APRIL secretion, suggesting the requirement for sulfation to induce this process. Indeed, except for non-sulfated hyaluronan, sulfation of other GAGs is crucial for their function. HS and CS are the primary GAGs involved in cell signaling and their sulfation status influences their protein interactions^26^ and signaling mechanisms^27,28^. It is unclear whether structural variation, mutation or the associated core protein component influences the BCC GAG’s ability to stimulate neutrophil APRIL secretion. While the HS-associated core proteins can act autonomously from the HS side chains^29^, the latter mostly determine the ligand-binding ability and hence the HSPG function^30^. Thus, although the core proteins present in various cell types may be similar, their unique HS side chains render discrete functions for HSPGs^30,31^, such as in growth factor regulation and cancer progression^32^. Interestingly, chondroitin sulfate proteoglycans (CSPG), which are expressed at higher levels in breast tumor tissues compared to normal breast tissues, promote metastasis by acting as a P-selectin ligand via the CS side chain^33^. In our studies, CS together with HS appears to contribute to breast cancer pathogenesis by stimulating secretion of the growth promoting APRIL from neutrophils that are recruited into the breast tumor microenvironment.

### TLR4 and PKR are involved in the breast cancer cell glycosaminoglycan-induced APRIL secretion from neutrophils

**Fig. 3:**
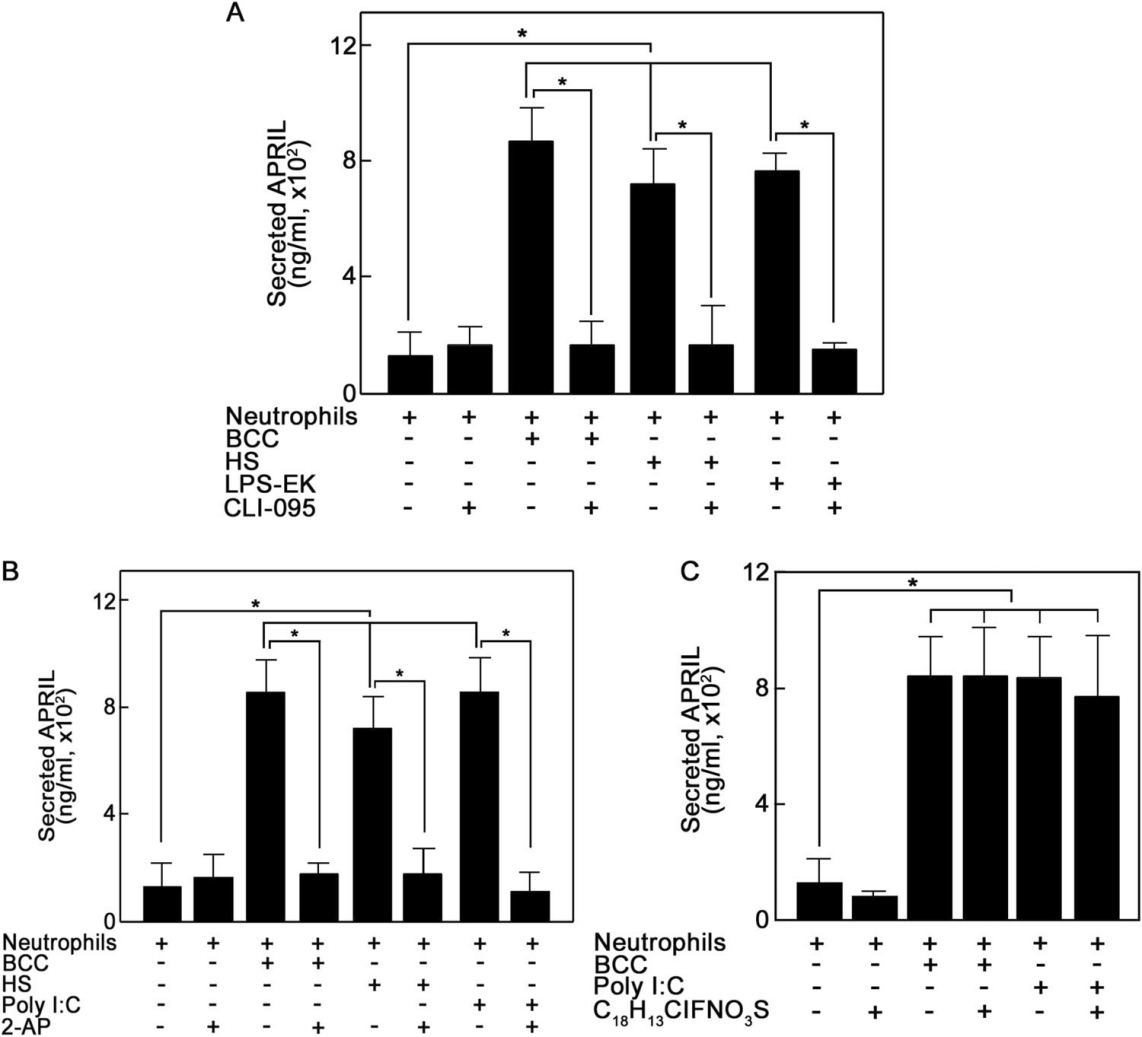
The glycosaminoglycan-mediated breast cancer cell-induced APRIL secretion from neutrophils utilizes the TLR4 pathway. To determine whether APRIL secretion involves the TLR4 pathway, potentially via PKR or TLR3, we took advantage of available biological/chemical activators and inhibitors. TLR4 and PKR were activated using ultra-pure lipopolysaccharide extracted from *E. coli* strain K12 (LPS-EK) and polyinosinic:polycytidylic acid (poly I:C), respectively. TLR4 was inhibited using a novel cyclohexene derivative, CLI-095. PKR and TLR3 were inhibited using 2-aminopurine (2-AP), and TLR3 was specifically inhibited using (*R*)-2-(3-Chloro-6-fluorobenzo[b]thiophene-2-carboxamido)-3-phenylpropanoic acid (C_18_H_13_ClFNO_3_S). Neutrophils (2 × 10^6^) were pre-treated (or untreated) with inhibitors [CLI-095 (5 mM; Invivogen), 2-AP (5 mM; Sigma), or C_18_H_13_ClFNO_3_S (10 μM; Calbiochem)] for 30 min at 37 °C prior to stimulation with T47D BCCs (5 × 10^5^), HS (10 μg/mL), LPS-EK (10 μg/mL; Invivogen) or poly I:C (25 μg/mL; Invivogen) for 5 min at 37 °C. Secreted neutrophil APRIL was measured by ELISA as described in Fig. legend 1. **a** TLR4 inhibition prevents BCC-, HS-, and LPS-EK-induced neutrophil APRIL secretion. Neutrophils were treated (or untreated) with the TLR4-specific inhibitor, CLI-095, prior to incubation with BCCs, HS or the TLR4-specific agonist, LPS-EK. **b** The PKR and TLR3 inhibitor, 2-AP, prevents BCC-, HS- and poly I:C-induced neutrophil APRIL secretion. Neutrophils were treated (or untreated) with 2-AP then incubated with BCCs, HS or poly I:C. **c** The TLR3-selective inhibitor, C_18_H_13_ClFNO_3_S, does not prevent BCC- and poly I:C-induced neutrophil APRIL secretion. Neutrophils were treated (or untreated) with C_18_H_13_ClFNO_3_S then incubated with BCCs or poly I:C. Data are means ± SD from three replicates of a representative experiment; *n* = 4. One-tailed Student’s *t*-test was used to determine statistical significance at *p* < 0.05

HS activates TLR4 in dendritic cells, causing enhanced cell maturation and alloreactive T-cell responses^34^, and lipopolysaccharide (LPS), a TLR4-specific bacterial ligand, increases APRIL expression in Caco-2 intestinal epithelial cells^35^. In neutrophils treated with LPS, TLR4 expression positively correlates with APRIL expression^36^, and this was suggested to play a part in the TLR4-mediated host immune response. Here, we examined the possibility that TLR4 is involved in BCC HS-induced neutrophil APRIL secretion. To do so, we took advantage of the TLR4-specific agonist, LPS-EK, and the TLR4-specific inhibitor, CLI-095. Figure 3a shows that LPS-EK induced neutrophil APRIL secretion to an extent similar to those induced by BCCs and HS, and pre-treatment of neutrophils with CLI-095 completely inhibited LPS-EK-induced secretion as well as secretion induced by BCCs and HS, suggesting that the BCC GAG-induced neutrophil APRIL secretion involves the TLR4 pathway. It is interesting that bacterial sensing in intestinal epithelial cells, which occurs through LPS activation of TLR4, causes APRIL-induced IgA2 class switching in lamina propria B cells^35^. In the breast tumor microenvironment, neutrophil APRIL appears to promote breast cancer progression as we (unpublished observation) and others^23^ found that APRIL induces BCC growth.

In vascular smooth muscle cells, heparin, a highly sulfated form of HS, activates PKR that blocks proliferation by inhibiting the cell cycle G1-S transition^37^. Since PKR can act downstream of TLR4^38^, we investigated whether BCC HS-induced neutrophil APRIL secretion also involves PKR. As shown in Fig. 3b, the PKR activator, poly I:C, induced neutrophil APRIL secretion to an extent similar to those induced by BCCs and HS. Pre-treatment of neutrophils with the potent PKR inhibitor, 2-AP, completely prevented poly I:C-, BCC-, and HS-induced APRIL secretion, suggesting PKR involvement in BCC HS-induced neutrophil APRIL secretion.

Since poly I:C also activates TLR3^39^, which regulates IL-32 and IFN-β secretion^40^, we tested whether TLR3 is also involved in BCC-induced neutrophil APRIL secretion. As observed above, poly I:C and BCCs induced similar extents of neutrophil APRIL secretion (Fig. 3c). However, pre-treatment of neutrophils with the TLR3-selective inhibitor, C_18_H_13_ClFNO_3_S, did not prevent poly I:C- or BCC-induced APRIL secretion, suggesting that TLR3 is not involved in the process, and that poly I:C and BCC activation of PKR, and not of TLR3, triggers secretion. Thus, while PKR can mediate functions regulated by TLR3 and TLR4 signaling^41^, and PKR can act downstream of these TLRs^38,42^, our findings suggest that PKR-mediated BCC GAG-induced neutrophil APRIL secretion occurs through TLR4 and not TLR3. As with HS and CS, the amounts of LPS-EK and poly I:C used in this study were sufficient to elicit secretion as much as that induced by BCCs. As indicated above, this may reflect the maximum amount of secretable APRIL from the specified number of neutrophils used. Nonetheless, measured secretion is sufficient to determine the significance of TLR4 and PKR in BCC GAG-induced neutrophil APRIL secretion.

### The TLR4 pathway leading to neutrophil APRIL secretion requires PKR

**Fig. 4:**
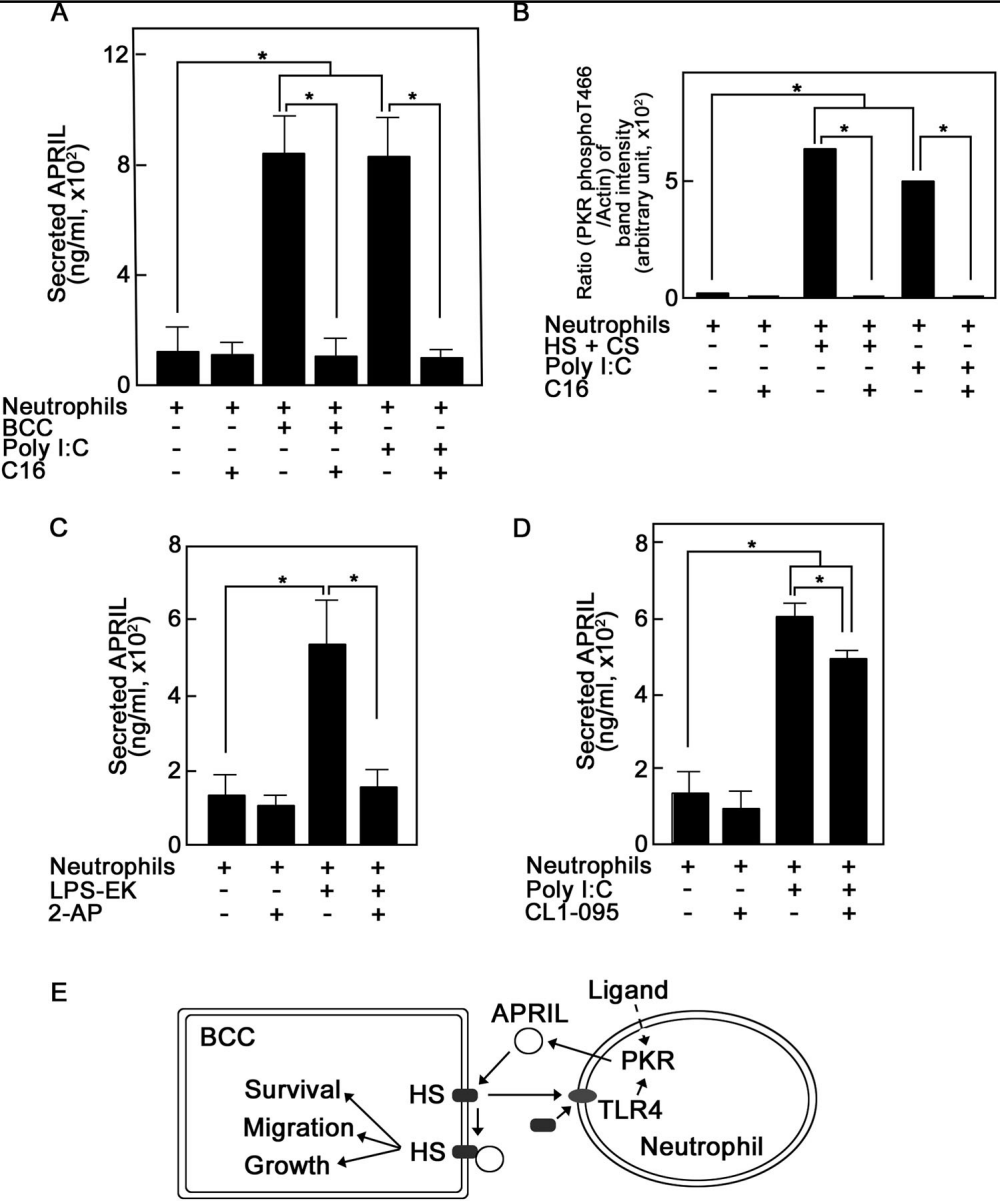
PKR is required for breast cancer cell glycosaminoglycan-induced APRIL secretion from neutrophils. **a** Specific inhibition of PKR prevents BCC- and poly I:C-induced neutrophil APRIL secretion. Neutrophils (2 × 10^6^) were pre-treated (or untreated) with the imidazolo-oxindole PKR-specific inhibitor, C16 (1 μM; Sigma), then incubated with BCCs (5 × 10^5^) or the PKR activator, poly I:C (25 μg/mL) for 5 min at 37 °C. **b** Specific inhibition of PKR prevents HS and CS glycosaminoglycan- and poly I:C-induced neutrophil PKR phosphorylation at T446. Neutrophils (2 × 10^6^) were pre-treated (or untreated) with C16 (1 μM) then incubated with HS (10 μg/mL) and CS (20 μg/mL) or poly I:C (25 μg/mL) for 30 s at 37 °C. Neutrophils were immediately pelleted and lysed in 25 mM HEPES containing 1 mM EDTA, 250 mM NaCl, 5 mM DTT, 10 mM NaF, 1 mM Na_3_VO_4_, 1% Triton X-100, 1 mM PMSF, and 1 μg/mL each of leupeptin, antipain, and aprotinin. Neutrophil lysates were subjected to SDS-PAGE then western blotting using anti-PKR phospho-T446 (Abcam) or -actin (C-19, Santa Cruz Biotech.). Immunoreactive bands were detected using ECL detection reagents (GE Health Care) and analyzed by densitometry using NIH ImageJ 1.61. Densitometry ratios of PKR phospho-T446/actin bands from a representative experiment using neutrophils from the same donor are shown. **c** The PKR inhibitor, 2-AP, prevents neutrophil APRIL secretion resulting from TLR4 activation by LPS-EK. Neutrophils (2 × 10^6^) were pre-treated (or untreated) with 2-AP (5 mM), then incubated with the TLR4-specific activator, LPS-EK (10 μg/mL) for 5 min at 37 °C. **d** TLR4 inhibition reduces neutrophil APRIL secretion resulting from PKR activation by poly I:C. Neutrophils (2 × 10^6^) were pre-treated (or untreated) with the TLR4-specific inhibitor, CLI-095 (5 mM), then incubated with poly I:C (25 μg/mL) for 5 min at 37 °C. In **a**–**d**, neutrophils were pre-treated with inhibitors for 30 min at 37 °C prior to stimulation with BCCs, poly I:C, HS and CS or LPS-EK. Secreted neutrophil APRIL was measured by ELISA as described in Fig. legend 1. Values are means ± SD from three replicates of a representative experiment where *n* = 3. Statistical significance was determined using one-tailed Student’s *t*-test (*p* < 0.05). **e** Schematic diagram showing the dual role of HS as a cell surface receptor or docking molecule for APRIL and as a ligand that induces neutrophil APRIL secretion via the TLR4-PKR pathway

To further investigate PKR involvement in neutrophil APRIL secretion, we utilized the imidazolo-oxindole PKR-specific inhibitor, C16. As shown in Fig. 4a, C16 completely prevented BCC- and poly I:C-induced neutrophil APRIL secretion, further indicating the requirement for PKR in BCC-induced secretion. The human PKR consists of about 15 autophosphorylation sites but only the activation loop T446 and T451 are crucial for kinase activity^43^. Figure 4b shows that neutrophil PKR T446 autophosphorylation significantly increased upon treatment with HS and CS or poly I:C, and this autophosphorylation was completely inhibited by C16, indicating that PKR activation is involved in BCC GAG-induced neutrophil APRIL secretion.

Since our findings indicate that both TLR4 and PKR are involved in BCC GAG-induced neutrophil APRIL secretion, we next examined a link between TLR4 and PKR. As with our findings above, Fig. 4c shows that TLR4 activation by LPS-EK-induced neutrophil APRIL secretion. Inhibition of neutrophil PKR using 2-AP prevented the LPS-EK-induced TLR4-mediated APRIL secretion, suggesting PKR requirement in this process. This is consistent with the finding in dendritic cells where regulation of APRIL secretion by specific TLR ligands depends on PKR^44^. Conversely, in Fig. 4d, we show that although inhibition of TLR4 using CLI-095 significantly reduced secretion stimulated by PKR activation using poly I:C, the TLR4-independent PKR-mediated secretion was significant and indicates that PKR activation is sufficient to induce APRIL secretion. This observation conforms with studies suggesting that the PKR pathway can be TLR-dependent or -independent, depending on the cell and functional system that is being examined^45,46^. In BCC GAG-induced neutrophil APRIL secretion, it is clear that both TLR4 and PKR are required to induce the process as separate and specific inhibition of TLR4 (Fig. 3a) or PKR (Fig. 4a) completely prevented secretion. The fact that specific TLR4 inhibition completely blocks PKR-mediated BCC GAG-induced secretion is consistent with the demonstrated downstream role of PKR in TLR-induced signaling^38^. Thus, we demonstrate that neutrophil TLR4 and PKR are targets of BCC GAGs and effectors of BCC GAG-induced neutrophil APRIL secretion. HS-induced TLR4-mediated secretion likely occurs via the TLR4 RHIFWRR extracellular domain, the HS binding motif^47^. Further molecular links between BCC GAG activation of TLR4 and PKR, and subsequent APRIL secretion by neutrophils remain to be explored.

In summary, we propose a model (Fig. 4e) whereby on top of the recognized role of HS in binding APRIL^3,8^, resulting in survival and migration signals^3^ and tumor growth^8^, HS (as well as CS), through the TLR4-PKR pathway, promotes APRIL secretion from neutrophils, demonstrating a novel link between HS and APRIL. The dual role of HS as cell surface receptor or docking molecule for APRIL and as a ligand that induces neutrophil APRIL secretion may be utilized by BCCs to enhance their own growth. Our premise is that BCCs take advantage of the neutrophil immune response and recruitment to tumor tissues to promote breast cancer progression.
